# Supplementary material for: Synergistic effect of nano-selenium and metformin on type 2 diabetic rat model: Diabetic complications alleviation through insulin sensitivity, oxidative mediators and inflammatory markers
Source: PLoS One. 2019 Aug 23;14(8):e0220779. doi: 10.1371/journal.pone.0220779 (PMC6707613; doi:10.1371/journal.pone.0220779)
Supplement: S2 Data — (PDF) [file pone.0220779.s002.pdf]

S2 Table 1. Evaluation of antioxidant activities of Se-NPs. Values are related to Fig. 3 (A-E) and represented as mean ± SE.

**Se concentration mg/ml**	**DPPH radical scavenging activity %**		**% inhibition of NO production**		**Hydrogen peroxide scavenging activity %**		**TAC %**		**Reducing Power assay %**	
	Se-NPs	Ascorbic acid	Se-NPs	Ascorbic Acid	Se-NPs	Ascorbic Acid	Se-NPs	Ascorbic Acid	Se-NPs	Ascorbic Acid
**1**	23±1.03	82±0.78	25±0.89	80±1.18	21±0.75	80±0.87	25±0.40	83±0.48	22±0.36	81±0.26
**2**	37±1.38	88±1.58	39±0.82	85±1.39	36±1.32	83±1.10	39±0.31	87±0.42	35±0.34	85±0.34
**3**	45±1.29	94±1.03	58±1.35	89±0.72	41±1.21	85±1.30	58±0.21	90±0.35	48±0.37	89±0.20
**4**	68±1.37	95±1.17	73±1.50	92±1.20	56±1.23	90±2.39	70±0.29	95±0.42	62±0.42	90±0.33
**5**	81±0.97	97±0.97	84±1.17	95.4±0.94	77±0.26	93±0.59	89±0.26	97±0.21	83±0.51	94±0.54

S2 Table 2. Changes in body weight of control groups fed with normal diet through 16 weeks. Values are mean ± SE (n = 10).

Weeks/Groups	Control	Se-NPs (0.1mg)	Se-NPs (0.4 mg)	MET	Se-NPs (0.1mg) +MET	Se-NPs (0.4 mg) +MET
**1**	100±1.35	90±0.95	98±1.45	108±2.23	103±0.93	100±1.22
**2**	115±1.54	102±1.22	110±3.50	112±2.56	115±1.55	111±1.95
**3**	125±1.26	115±1.54	122±2.44	120±1.45	126±3.54	123±3.77
**4**	140±1.23	125±1.20	130±5.52	135±1.89	136±1.34	133±2.04
**5**	165±1.56	137±2.30	145±1.67	142±4.66	149±2.22	145±3.06
**6**	184±1.62	149±1.54	157±1.35	153±3.65	159±1.09	155±5.53
**7**	200±2.40	161±1.44	169±6.55	159±1.40	170±3.65	166±1.45
**8**	205±1.50	179±2.70	180±4.67	164±2.23	188±7.33	175±3.30
**9**	215±2.10	185±5.77	194±3.56	170±1.88	200±1.59	191±2.54
**10**	220±1.99	201±8.34	215±1.25	180±5.65	215±3.44	201±5.03
**11**	230±1.37	215±3.56	225±2.88	196±3.56	224±2.45	217±1.45
**12**	241±2.44	232±6.77	239±7.70	205±3.44	237±1.44	225±1.87
**13**	256±1.33	247±2.56	247±3.56	210±2.69	244±1.38	236±2.45
**14**	269±2.70	258±2.89	255±2.57	222±1.34	259±8.44	247±2.77
**15**	280±2.60	263±4.78	269±3.50	234±1.09	264±3.54	258±6.67
**16**	291±1.56	284±3.67	289±1.44	240±3.04	281±3.09	268±3.80

S2 Table 3. Changes in body weight of HFD/STZ-induced rats and after Se-NPs and MET treatment through 16 weeks. Values are mean ± SE (n = 10).

Weeks/ Groups	HFD/STZ	Se-NPs (0.1mg) Trx	Se-NPs (0.4mg) Trx	MET Trx	Se-NPs (0.1mg) +MET Trx	Se-NPs (0.4mg) +MET Trx
**1**	110±0.89	118±1.10	107±1.10	113±2.17	107±1.23	95±0.99
**2**	130±0.99	126±1.34	123±2.11	140±1.33	125±1.33	119±2.31
**3**	150±2.45	146±2.44	137±2.04	165±1.09	149±2.45	139±1.76
**4**	200±1.50	170±1.38	150±1.74	194±3.01	166±0.95	175±1.43
**5**	230±2.44	205±1.45	170±2.22	240±2.55	184±2.10	201±1.00
**6**	274±1.65	230±1.90	207±1.06	280±2.65	216±2.54	230±2.43
**7**	300±1.77	255±1.56	230±0.95	317±3.53	236±1.78	254±2.41
**8**	345±2.78	290±1.88	260±1.19	333±4.03	270±1.95	284±1.32
**9**	330±2.67	309±1.60	269±1.35	340±1.52	274±3.03	292±1.33
**10**	311±1.67	319±2.33	280±2.45	345±1.04	281±1.10	298±2.44
**11**	293±1.48	332±2.74	297±2.44	345±2.66	287±1.00	305±2.76
**12**	284±3.66	344±3.11	301±1.47	350±1.45	290±2.45	315±3.70
**13**	277±2.74	353±2.76	320±2.09	353±1.34	298±2.77	321±1.73
**14**	270±2.55	358±1.94	325±1.10	358±1.75	301±2.00	327±1.99
**15**	265±1.53	363±2.40	331±2.35	360±2.85	310±1.87	332±2.04
**16**	254±2.09	369±2.66	340±3.44	366±1.94	315±1.98	335±2.77

S2 Table 4. AGEs levels on serum and liver tissues of HFD/STZ-induced rats and after treatment with Se-NPs and MET. Values are mean ± SE (n = 10). Significant changes (p<0.01) with respect to control and HFD/STZ-experimental rats are expressed by the letters (a) and (b), respectively.

Groups/Parameters	Serum (U/ml)	Liver (U/mg protein)
**Control**	20.2±0.77[b]	3.2±0.13[b]
**Se-NPs (0.1mg)**	14.4±0.84[b]	2.5±0.25[b]
**Se-NPs (0.4 mg)**	9.09±0.94[b]	2.01±0.25[b]
**MET**	13.5±0.62[b]	3.9±0.19[b]
**Se-NPs (0.1mg)+MET**	18.7±0.93[b]	1.8±0.98[a, b]
**Se-NPs (0.4 mg)+MET**	7.93±0.83[b]	1.1±0.08[a, b]
**HFD/STZ**	97.3±1.98[a]	19.55±0.31[a]
**Se-NPs (0.1mg) Trx**	25.09±0.91[a, b]	4.9±0.18[a, b]
**Se-NPs(0.4mg) Trx**	21.03±0.71[b]	4.1±0.18[b]
**MET Trx**	30.4±0.79[a, b]	3.3±0.24[b]
**Se-NPs (0.1mg)+MET Trx**	19.23±0.93[b]	2.29±0.83[b]
**Se-NPs (0.4mg)+MET Trx**	15.9±0.93[b]	1.49±0.80[a, b]

S2 Table 5. Serum and hepatic inflammatory markers in HFD/STZ-induced rats treated with Se-NPs and MET. Values are mean ± SE (n = 10). Significant changes ($p<0.01$) with respect to control and HFD/STZ-experimental rats are expressed by the letters (a) and (b), respectively.

**Groups/Parameters**	**iNOS**		**TNF-α**		**IL-6**		**IL-1β**	
	Liver (ng/mg protein)	Serum (ng/ml)	Liver (pg/mg protein)	Serum (pg/ml)	Liver (pg/mg protein)	Serum (pg/ml)	Liver (pg/mg protein)	Serum (pg/ml)
**Control**	4.7±0.59 [b]	3.4±0.20 [b]	5.8±0.59 [b]	6.1±0.30 [b]	20.3±0.33 [b]	17.4±0.39 [b]	5.9±0.48 [b]	4.3±0.34 [b]
**SeNPs (0.1mg)**	3.8±0.13 [b]	2.2±0.10 [b]	3.4±0.13 [b]	4.9±0.09 [b]	17.3±0.13 [b]	15.8±0.29 [b]	4.6±0.34 [b]	3.6±0.45 [b]
**SeNPs (0.4 mg)**	3.1±0.23 [b]	2.0±0.40 [b]	3.07±0.11 [b]	4±0.20 [b]	15.2±0.45 [b]	14.2±0.23 [b]	4.1±0.56 [b]	3.2±0.40 [b]
**MET**	4.1±0.17 [b]	2.5±0.70 [b]	3.7±0.17 [b]	5.1±0.07 [b]	18.3±0.80 [b]	13.9±0.26 [b]	5.1±0.40 [b]	4.2±0.70 [b]
**SeNPs (0.1mg)+MET**	2.9±0.38 [a, b]	1.98±0.71 [a, b]	2.1±0.45 [a, b]	3.6±0.12 [a, b]	10.2±0.50 [a, b]	11.8±0.28 [a, b]	3.7±0.23 [a, b]	2.9±0.34 [a, b]
**SeNPs (0.4 mg)+MET**	2.1±0.28 [a, b]	1.02±0.30 [a, b]	1.4±0.10 [a, b]	2.8±0.32 [a, b]	8.4±0.30 [a, b]	9.2±0.30 [a, b]	2.9±0.34 [a, b]	1.3±0.40 [a, b]
**HFD/STZ**	31.03±0.90 [a]	18.9±0.50 [a]	44.5±1.2 [a]	35.3±0.95 [a]	111.8±1.30 [a]	95.4±0.99 [a]	30.5±0.98 [a]	23.5±0.99 [a]
**SeNPs (0.1mg) Trx**	12.3±0.29 [a]	4.67±0.38 [a, b]	12.3±0.23 [a, b]	18.3±0.44 [a, b]	23.4±0.66 [a, b]	27.8±0.50 [a, b]	15.2±0.30 [a, b]	11.8±0.40 [a, b]
**SeNPs(0.4mg) Trx**	10.3±0.30 [a]	4.1±0.59 [a, b]	10.2±0.21 [a, b]	17.1±0.23 [a, b]	21.3±0.40 [a, b]	25.1±0.34 [a, b]	13.9±0.40 [a, b]	10.03±0.60 [a, b]
**MET Trx**	13.01±0.14 [a]	5.11±0.28 [a, b]	13.9±0.44 [a, b]	18.6±0.52 [a, b]	27.3±0.44 [a, b]	33.9±0.50 [a, b]	17.4±0.60 [a, b]	13.5±0.40 [a, b]
**SeNPs (0.1mg)+MET Trx**	7.23±0.33 [a]	3.98±0.23 [a, b]	7.3±0.34 [a, b]	16.1±0.45 [a, b]	19.2±0.50 [b]	21.8±0.30 [b]	12.3±0.51 [a, b]	8.7±0.38 [a, b]
**SeNPs (0.4mg)+MET Trx**	4.67±0.31 [b]	3.03±0.34 [b]	5.7±0.37 [b]	10.1±0.34 [a, b]	17.99±0.55 [b]	18.2±0.32 [b]	9.3±0.31 [a, b]	6.1±0.36 [a, b]

S2 Table 6. Western blot analysis of hepatic inflammatory markers in HFD/STZ-induced rats treated with Se-NPs and MET. Values are mean ± SE (n = 3). Significant changes ($p<0.01$) with respect to control and HFD/STZ-experimental rats are expressed by the letters (a) and (b), respectively.

Groups/Parameters	COX2/β-actin	p-p65/ β-actin
**Control**	1.00±0.01 [b]	1.09±0.02 [b]
**Se-NPs (0.1mg)**	1.03±0.03 [b]	1.29±0.03 [b]
**Se-NPs (0.4 mg)**	0.93±0.02 [b]	0.63±0.02 [a, b]
**MET**	0.90±0.05 [b]	1.22±0.04 [b]
**Se-NPs (0.1mg)+MET**	0.42±0.02 [a, b]	0.26±0.05 [a, b]
**Se-NPs (0.4 mg)+MET**	0.34±0.04 [a, b]	0.45±0.02 [a, b]
**HFD/STZ**	2.80±0.01 [a]	4.10±0.06 [a]
**Se-NPs (0.1mg) Trx**	1.14±0.03 [b]	0.88±0.03 [a, b]
**Se-NPs(0.4mg) Trx**	1.14±0.04 [b]	0.74±0.01 [a, b]
**MET Trx**	1.04±0.05 [b]	0.65±0.02 [a, b]
**Se-NPs (0.1mg)+MET Trx**	0.88±0.02 [a, b]	0.55±0.04 [a, b]
**Se-NPs (0.4mg)+MET Trx**	0.69±0.02 [a, b]	0.58±0.03 [a, b]

S2 Table 7. Hepatic mRNA expression of inflammatory genes in control and HFD/STZ-induced rats treated with Se-NPs and MET. Values are mean ± SE (n = 3). Significant changes ($p<0.01$) with respect to HFD/STZ-experimental rats are expressed by the letters (b).

Groups/Parameters	IL-23	IL-1α	mcp-1	Socs-3	Ox-LDL
**Se-NPs (0.1mg)**	1.988±0.22 ^{b}	1.1±0.19 ^{b}	0.76±0.20 ^{b}	1.1±0.17 ^{b}	0.34±0.21 ^{b}
**Se-NPs (0.4 mg)**	1.2±0.21 ^{b}	0.8±0.20 ^{b}	0.24±0.14 ^{b}	0.73±0.19 ^{b}	0.22±0.23 ^{b}
**MET**	1.3±0.10 ^{b}	1.3±0.18 ^{b}	0.75±0.23 ^{b}	0.98±0.21 ^{b}	0.45±0.19 ^{b}
**Se-NPs (0.1mg)-MET**	0.2±0.13 ^{b}	-0.3±0.11 ^{b}	-2.3±0.09 ^{b}	0.2±0.10 ^{b}	0.04±0.05 ^{b}
**Se-NPs (0.4 mg)-MET**	-2.5±0.01 ^{b}	-1.3±0.02 ^{b}	-5.8±0.01 ^{b}	-0.94±0.01 ^{b}	-1.1±0.02 ^{b}
**HFD/STZ**	23.75±0.54	29.76±0.65	24.36±0.45	18.54±0.43	25.44±0.55
**Se-NPs (0.1mg) Trx**	5.3±0.23 ^{b}	8.9±0.34 ^{b}	8.7±0.21 ^{b}	3.2±0.12 ^{b}	7.2±0.23 ^{b}
**Se-NPs(0.4mg) Trx**	3.2±0.24 ^{b}	7.4±0.21 ^{b}	7.3±0.13 ^{b}	3±0.23 ^{b}	5.3±0.24 ^{b}
**MET Trx**	5.5±0.22 ^{b}	8.8±0.17 ^{b}	7.7±0.23 ^{b}	3.4±0.22 ^{b}	7.7±0.29 ^{b}
**Se-NPs (0.1mg)-MET Trx**	2.22±0.25 ^{b}	2.3±0.22 ^{b}	3.2±0.25 ^{b}	2.5±0.29 ^{b}	2.1±0.17 ^{b}
**Se-NPs (0.4mg)-MET Trx**	0.997±0.21 ^{b}	1.2±0.24 ^{b}	1.4±0.22 ^{b}	1.2±0.23 ^{b}	0.94±0.15 ^{b}

S2 Table 8. Western blot analysis of hepatic insulin signaling pathway in HFD/STZ-induced rats treated with Se-NPs and MET. Values are related to Fig. 3 A-E and represented as mean ± SE (n = 3). Significant changes (p<0.01) with respect to control and HFD/STZ-experimental rats are expressed by the letters (a) and (b), respectively.

Groups/Parameters	p-IRS/IRS	p-AKT/AKT	p-GSK-3β/GSK-3β	p-AMPK/AMPK
**Control**	0.64±0.008 [b]	0.57±0.01 [b]	0.64±0.02 [b]	0.46±0.01 [b]
**Se-NPs (0.1mg)**	0.88±0.009 [a, b]	0.68±0.009 [b]	1.12±0.03 [a, b]	0.89±0.03 [a, b]
**Se-NPs (0.4 mg)**	1.82±0.01 [a, b]	1.27±0.02 [a, b]	1.06±0.01 [a, b]	0.77±0.02 [a, b]
**MET**	1.08±0.03 [a, b]	0.65±0.01 [b]	1.49±0.009 [a, b]	1.03±0.03 [a, b]
**Se-NPs (0.1mg)+MET**	1.17±0.02 [a, b]	1.28±0.03 [a, b]	1.00±0.01 [a, b]	1.59±0.04 [a, b]
**Se-NPs (0.4 mg)+MET**	1.58±0.01 [a, b]	1.69±0.01 [a, b]	1.50±0.01 [a, b]	2.33±0.03 [a, b]
**HFD/STZ**	0.28±0.003 [a]	0.23±0.007 [a]	0.17±0.009 [a]	0.25±0.007 [a]
**Se-NPs (0.1mg) Trx**	0.90±0.03 [a, b]	0.76±0.02 [a, b]	0.85±0.03 [a, b]	0.66±0.01 [a, b]
**Se-NPs(0.4mg) Trx**	0.57±0.009 [a, b]	0.96±0.01 [a, b]	0.95±0.01 [a, b]	0.72±0.01 [a, b]
**MET Trx**	0.89±0.01 [a, b]	1.19±0.04 [a, b]	1.08±0.02 [a, b]	0.90±0.02 [a, b]
**Se-NPs (0.1mg)+MET Trx**	0.94±0.02 [a, b]	1.40±0.03 [a, b]	1.19±0.04 [a, b]	0.97±0.02 [a, b]
**Se-NPs (0.4mg)+MET Trx**	1.48±0.02 [a, b]	1.42±0.01 [a, b]	1.08±0.01 [a, b]	1.45±0.01 [a, b]
