# Supplementary material for: Synergistic effect of nano-selenium and metformin on type 2 diabetic rat model: Diabetic complications alleviation through insulin sensitivity, oxidative mediators and inflammatory markers
Source: PLoS One. 2019 Aug 23;14(8):e0220779. doi: 10.1371/journal.pone.0220779 (PMC6707613; doi:10.1371/journal.pone.0220779)
Supplement: Attachment — Submitted filename: Response to reviewing.docx [file pone.0220779.s003.docx]

Dear Editor

I will be very grateful if you consider the revised manuscript entitled “Synergistic effect of nano-selenium and metformin on type 2 diabetic rat model: Diabetic complications alleviation through insulin sensitivity, oxidative mediators and inflammatory markers” for the publication in “PLOS ONE”. The response to journal requirements and reviewers comments (yellow-labeled) was done point by point as seen below. The authors appreciate the valuable comments by the reviewers.

Regards.

Sincerely

The Corresponding Author

Prof Dr. Mahmoud Balbaa

**Journal Requirements:**

When submitting your revision, we need you to address these additional requirements.

Please ensure that your manuscript meets PLOS ONE's style requirements, including those for file naming. The PLOS ONE style templates can be found at

http://www.journals.plos.org/plosone/s/file?id=wjVg/PLOSOne_formatting_sample_main_body.pdf and http://www.journals.plos.org/plosone/s/file?id=ba62/PLOSOne_formatting_sample_title_authors_affiliations.pdf

1. In your Material and Methods section we note that you state the following "All animal studies were performed according to the animal protocols approved by the Institutional Ethics Committee of Faculty of Science, Alexandria University, Egypt in accordance with the ethical standards of the Helsinki Declaration of 1975, as revised in 2008." The Declaration of Helsinki is a set of ethical principles regarding the use of human participants in experimental or medical research. We understand that the submitted work only uses animals and we would therefore kindly ask that you remove the reference fo the Helsinki Declaration.

[Note: HTML markup is below. Please do not edit.]

**Corrected**

Reviewers' comments:

Reviewer's Responses to Questions

Comments to the Author

1. Is the manuscript technically sound, and do the data support the conclusions?

The manuscript must describe a technically sound piece of scientific research with data that supports the conclusions. Experiments must have been conducted rigorously, with appropriate controls, replication, and sample sizes. The conclusions must be drawn appropriately based on the data presented.

Reviewer #1: Yes

Reviewer #2: Partly

2. Has the statistical analysis been performed appropriately and rigorously?

Reviewer #1: Yes

Reviewer #2: Yes

3. Have the authors made all data underlying the findings in their manuscript fully available?

The PLOS Data policy requires authors to make all data underlying the findings described in their manuscript fully available without restriction, with rare exception (please refer to the Data Availability Statement in the manuscript PDF file). The data should be provided as part of the manuscript or its supporting information, or deposited to a public repository. For example, in addition to summary statistics, the data points behind means, medians and variance measures should be available. If there are restrictions on publicly sharing data—e.g. participant privacy or use of data from a third party—those must be specified.

Reviewer #1: Yes

Reviewer #2: Yes

4. Is the manuscript presented in an intelligible fashion and written in standard English?

PLOS ONE does not copyedit accepted manuscripts, so the language in submitted articles must be clear, correct, and unambiguous. Any typographical or grammatical errors should be corrected at revision, so please note any specific errors here.

Reviewer #1: No

Reviewer #2: No

**The whole file of the manuscript was subjected to intensive language revision for the contextual spelling, grammar and sentence structure. Minor mistakes were found and corrected (grey-labeled).**

**5.** **Review Comments to the Author**

Please use the space provided to explain your answers to the questions above. You may also include additional comments for the author, including concerns about dual publication, research ethics, or publication ethics. (Please upload your review as an attachment if it exceeds 20,000 characters)

Reviewer #1: This work prepared selenium nanoparticles and investigated their antidiabetic action and synergy with metformin. The study is sufficient and sound in experiments and results. It provides insight into the use of selenium nanoparticles (Se-NPs) to combat type 2 DM. However, some minor revisions are required to be addressed for improving the manuscript. Here, I raised some recommendations to the authors for reference.

1. The title of this article needs to be amended. Type 2 diabetes-induced rats is a wrong expression. HFD/STZ-induced type 2 diabetes rats or type 2 diabetic model rats are more suitable.

**Changed**

2. The functions of selenium as antidiabetic agent should be elaborated in the introduction section. The authors can refer to the latest publication (Guan, B. et al. Selenium as a pleiotropic agent for medical discovery and drug delivery. Int. J. Nanomedicine. 2018, 13: 7473-7490) for citation.

**This part was inserted to introduction section as one paragraphs in pages 4.**

3. In the introduction section, some representative examples using selenium nanoparticles for treatment of diabetes should be contained to highlight the antidiabetic advantages of Se-NPs, for example, Deng, W. et al. Selenium nanoparticles as versatile carriers for oral delivery of insulin: Insight into the synergic antidiabetic effect and mechanism. Nanomedicine. 2017, 13: 1965-1974. Deng, W. et al. Selenium-layered nanoparticles serving for oral delivery of phytomedicines with hypoglycemic activity to synergistically potentiate the antidiabetic effect. Acta Pharmaceutica Sinica B. 2019, 9: 74-86.

**This part was inserted to introduction section in the paragraph of pages 4 & 5.**

4. Regarding the preparation of Se-NPs, why does the coating with dextrin happen after Se-NPs preparation. As a stabilizing agent, polymer is normally concurrently added into the system upon reaction of ionic selenium and reductant.

**In our study, the Se-NPs were coated with 5% dextrin by using a single layer coating method on a magnetic stirrer at ambient temperature instead of adding water. The nanoparticles were diluted by using a dextrin-containing solution and the samples were then washed and dried to increase the particle stability in solutions. In addition, the coating increases the viscosity of the medium, which in turn decreases the interaction between the particles present in the medium and prevent the Se-NPs precipitation. At the refrigeration temperature (4ᵒC), the coated Se-NPs were stable up to 1 year. The preparation and coating of Se-NPs were prepared according to the method described by (Qian Li et al, 2010) with some modifications**

**References:**

1) **Qian Li, Tianfeng Chen, Fang Yang, etal. Facile and controllable one-step fabrication of selenium nanoparticles assisted by L-cysteine. Materials Letters, 2010; 64: 614–617.**
2) **CHAPTER 3 Synthesis, Coating, Characterisation and Stability of Selenium Nanoparticles.**
3) **SONAM MALHOTRA, NEETU JHA & KRUTIKA DESAI. A superficial synthesis of selenium nanospheres using wet chemical approach. International Journal of Nanotechnology and Application (IJNA), 2014; 3: 7-14**
4) **Malhotra S, Welling MN, Mantri SB, Desai K: In vitro and in vivo antioxidant, cytotoxic, and anti-chronic inflammatory arthritic effect of selenium nanoparticles. J Biomed Mater Res B Appl Biomater, 2016; 104: 993–1003.**

5. Why is the insulin concentration in HFD/STZ model far higher than the control? Does HFD/STZ induction result in more secretion of insulin? This is abnormal that the insulin level does not reduce but inversely increase in diabetic rats in logicality.

**HFD/STZ induction increases the insulin resistance in which more insulin is not recognized with insulin receptor and therefore insulin will be secreted in serum compared to control.**

**An example of a chemical-induced animal model of diabetes is the HFD/STZ animal model. This model involves a combination of an HFD to bring about hyperinsulinemia, IR, and/or glucose intolerance followed by subsequent injection of a low dose 35 mg/kg STZ, which results in severe reduction in functional-cell mass. The transition from a metabolically healthy state to prediabetes often includes an obese state characterized by hyperinsulinemia, insulin resistance, and dyslipidemia. Further inflammation of the abdominal adipose tissue may worsen the dysfunctional state of the adipocytes, leading to more ectopic fat accumulation, insulin resistance and hyperinsulinemia. Also, this may be due to inability of insulin to act properly on resistant tissues and this resulted in poor glucose utilization. So, β-cells are initially compensated for insulin resistance by elevating insulin secretion. Beside, multiple organs contribute to the development of peripheral insulin resistance via impaired biological response to insulin. Finally, the events leading to β-cell compensatory mechanisms and subsequent β-cell failure in type 2 diabetes involve lipotoxicity and/or glucolipotoxicity, insulin resistance, hyperinsulinemia, and stress, with a modest contribution from low-level inflammation.**

**References:**

1- **Søs Skovsø. Modeling type 2 diabetes in rats using high fat diet and streptozotocin. J Diabetes Investig. 2014 Jul; 5(4): 349–358.**
2- **Saleh S., El-Maraghy N., Reda E. and Barakat W. Modulation of Diabetes and Dyslipidemia in Diabetic Insulin-Resistant Rats by Mangiferin: Role of Adiponectin and TNF-α. An Acad Bras Cienc, 2014. 86(4): 1935-1948.**
3- **Jia-You Fang, Chih-Hung Lin, Tse-Hung Huang, and Shih-Yi Chuang. In Vivo Rodent Models of Type 2 Diabetes and Their Usefulness for Evaluating Flavonoid Bioactivity. Nutrients. 2019 Mar; 11(3): 530.**
4- **Anisha A. Gupte, Laurie J. Minze, Maricela Reyes, Yuelan Ren, Xukui Wang, Gerd Brunner, Mohamad Ghosn, Andrea M. Cordero-Reyes, Karen Ding, Domenico Pratico, Joel Morrisett, Zheng-Zheng Shi, Dale J. Hamilton, Christopher J. Lyon, and Willa A. Hsueh. High-Fat Feeding-Induced Hyperinsulinemia Increases Cardiac Glucose Uptake and Mitochondrial Function Despite Peripheral Insulin Resistance. Endocrinology. 2013 Aug; 154(8): 2650–2662.**
5- **Erin E. Mulvihill, Emma M. Allister, Brian G. Sutherland, Dawn E. Telford, Cynthia G. Sawyez, Jane Y. Edwards, Janet M. Markle, Robert A. Hegele, and Murray W. Huff. Naringenin Prevents Dyslipidemia, Apolipoprotein B Overproduction, and Hyperinsulinemia in LDL Receptor–Null Mice With Diet-Induced Insulin Resistance. Diabetes. 2009 Oct; 58(10): 2198–2210.**
6- **Balbaa M, El-Zeftawy M, Ghareeb D, Taha N, Mandour AW. Nigella sativa Relieves the Altered Insulin Receptor Signaling in Streptozotocin-Induced Diabetic Rats Fed with a High-Fat Diet. Oxid Med Cell Longev. 2016;2016:2492107.**

6. The toxicity of Se-NPs is a great concern for its biomedical application. The authors should investigate the cytotoxicity of Se-NPs using cell test or other techniques.

**The determination of the parameters of hepatic function (Table 2) and renal function (Table 3) are important toxicological parameters as explained in page 14 in the subtitle”** **Serum liver and kidney function parameters”. In addition, an experiment was run to investigate the cytotoxicity of Se-NPs in a hepatocyte cell line. This investigation was inserted in “Materials and Methods” in the subtitle “Cytotoxicity study” (page7 & 8) and “Results” in the subtitle “Cytotoxicity Analysis” (page 14). Also, it was discussed in the first paragraph in “Discussion” (page 19 & 20). Moreover, there is no detected hepatic and renal toxicity by Se-NPs in the tested animals as shown in Tables 3 &4.**

7. The English language of this manuscript should be enhanced. Some expressions are confused in style.

**The whole file of the manuscript was subjected to intensive language revision for the contextual spelling, grammar and sentence structure. Minor mistakes were found and corrected (grey-labeled).**

Reviewer #2: - There are many English errors. The work should be revised by an English native speaker.

**The whole file of the manuscript was subjected to intensive language revision for the contextual spelling, grammar and sentence structure. Minor mistakes were found and corrected (grey-labeled).**

- The authors can't use "dose-dependent" with two investigated doses only, at list the word used with three doses.

**Corrected**

**All the expressions of “dose-dependent” were deleted from the test, table legends and figure caption except what explained in page 21 in “Discussion”, reference 62.**

- "HFD was administered" should be "HFD was supplemented".

**Corrected in abstract and pages 9 & 10.**

- "different diabetic complication pathways" vague expression. In this study, the authors focused mainly on the anti-diabetic effect of Se.

**“insulin sensitivity, oxidative mediators and inflammatory markers” replaces the mentioned expression.**

- "After 16 weeks of daily oral administration" should be 8 instead of 16.

**Corrected**

**"After 8 weeks of daily oral administration"**

- Histopathology of liver and pancreas are highly recommended.

**Since our target project has focused on the study of different signaling and inflammatory parameters, the histopathological studies of liver and pancreas were not in our plan. The tissues were not kept in formalin for the histopathological studies and we will consider it in the future in an extended study.**

- Figure's 2 legend needs more explanation.

**Changed to**

**Fig. 2. Representative Transmission Electron Microscopy analysis of prepared Se-NPs showing their size and shape.**

- How many assays were performed to obtain the results those used to illustrate fig.3?

**Five assays were performed to determine In *vitro* antioxidant bioactivities of prepared Se-NPs**

1- **DPPH radical scavenging activity**
2- **Nitric oxide radical scavenging activity**
3- **Hydrogen peroxide scavenging activity**
4- **Total antioxidant capacity assay**
5- **Reducing power assay**

- Final BW (µg) ???

**Corrected as Final BW (g). It is the body weight at the end of the 16 weeks expressed in grams.**
